# ECOLOGY AND WILDLIFE: Pharmed Fish

**DOI:** 10.1289/ehp.117-a240

**Published:** 2009-06

**Authors:** Kellyn S. Betts

Results from a recent pilot study of the uptake of pharmaceuticals and personal care products (PPCPs) by fish in U.S. rivers have garnered considerable attention since they were published online 25 March 2009 ahead of print in *Environmental Toxicology and Chemistry*. The study, led by Baylor University chemistry professor C. Kevin Chambliss and environmental science professor Bryan Brooks, broke new ground by assessing chemical contamination of wild fish at a variety of sampling sites, screening a relatively large and diverse array of PPCPs.

The work focused on five rivers in Chicago, Dallas, Orlando, Phoenix, and West Chester (Pennsylvania) that receive waste−water directly from treatment facilities. All five rivers are considered “effluent−dominated” because the high volume of wastewater they receive continuously exposes their aquatic inhabitants to effluent−derived contaminants. “Such [worst−case] exposure scenarios are not represented in standardized toxicity tests used to assess ecological risk of PPCPs to aquatic life,” Chambliss and Brooks point out in their paper.

The researchers screened samples of fish fillet (muscle and skin) for 24 pharmaceuticals and 12 personal care products, while samples of fish liver were screened for pharmaceuticals only. The team found levels of 7 pharmaceuticals and 2 personal care products above method detection limits in the tissues analyzed (see table). This is the first report of the presence of the lipid regulator gem−fibrozil in U.S. wild fish.

The team explained that they chose the study sites because point source discharges from wastewater treatment facilities are “the most significant entry route for human pharmaceuticals into the aquatic environment.” However, recent work by Joakim Larsson, an assistant professor at Göteborg University, published in the April 2009 issue of *Regulatory Toxicology and Pharmacology* suggest pharmaceutical manufacturing plants in the developing world can release much higher quantities into the nearby environment. Larsson says the impact of wastewater from pharmaceutical manufacturers in the developed world is not yet clear, but a 19 April 2009 Associated Press story reports that unpublished data from the U.S. Geological Survey (USGS) and the U.S Environmental Protection Agency (EPA) show U.S. plants also may emit high concentrations of pharmaceuticals.

In 2005, Chambliss and Brooks were part of the first team to document substantial uptake of pharmaceuticals in fish, and experts agree their latest findings are consistent with the work conducted to date by a number of scientists. Moreover, the findings have inspired the EPA, which funded the research, to expand its investigation of PPCPs in fish under its National Rivers and Streams Assessment to obtain a more detailed national perspective, says Suzanne Rudzinski, deputy director of the EPA Office of Science and Technology. The agency is investigating whether the concentrations of pharmaceuticals present in fish are of concern for human health or for the fish themselves, she says.

The very low concentrations of pharmaceuticals in the fish flesh may translate into “good news for public health concerns,” says Shane Snyder, a project manager at the Southern Nevada Water Authority’s Applied Research and Development Center. “You’d have to eat tons of fish to come even close to the pharmaceutical levels we predict would be of concern to human health,” he says. He also points out that effluent−dominated waterways constitute an “amazing minority” of U.S. streams.

Dana Kolpin, a project chief with the USGS Toxic Substances Hydrology Program, is more cautious, however. “What if these compounds bioaccumulate in tissue or have unintended consequences from the complex mixtures of contaminants we’re exposed to?” he asks. Moreover, he says, “USGS research has shown that PPCPs are commonly present in streams across the United States, not just those that are truly effluent−dominated. While these results in effluent−dominated streams may be the extreme case, this will not be the only setting where such PPCPs will be found in fish tissue.”

Last year, Kolpin and colleagues reported in the 15 March 2008 issue of *Environmental Science & Technology* that earthworms can take up PPCPs from biosolids applied on land. Considered together, the research to date begs for further study into the potential impacts of such chemical mixtures on both aquatic and terrestrial organisms, he says. This is particularly true because researchers’ analytical methods allow them to look for only a few hundred of the thousands of PPCPs that might be in U.S. water bodies, adds Herb Buxton, coordinator for the USGS Toxic Substances Hydrology Program.

Comparing personal care products and pharmaceuticals, Snyder says the former may merit greater investigation—some of the concentrations documented by Chambliss and Brooks for these compounds are orders of magnitude higher than those seen for pharmaceuticals. For example, the highest uptake reported in the study was of the synthetic fragrance galaxolide, which showed a maximum concentration of 2,100 ng/g in fish fillets (sertraline, the pharmaceutical consistently found at the highest concentrations, was detected in fillet and liver at 19 and 545 ng/g, respectively). The research literature makes it “pretty clear that [personal care products] are bioaccumulating,” Snyder says, adding that people often spray fragrance compounds all over themselves.

Larsson points out that although drugs are specifically designed to interfere with biological systems, synthetic musk fragrances are not. “Thus, even if levels are higher of the musks, it is not clearcut that the risks are higher with this group of chemicals,” he says.

Potential concerns raised by the new study include possible impacts on aquatic life in light of growing evidence that very low−level exposure to pharmaceuticals can impact fish and other underwater dwellers, Brooks and Kolpin point out. In time, research on the effects of PPCP exposure may inspire scientists to come up with new ways to conduct ecologic risk assessments, Brooks predicts. “Risk assessments are based on traditional responses in fish, such as changes in growth or survival, that we may not see when they’re exposed to PPCPs,” points out Karen Kidd, a biologist with the University of New Brunswick’s Canadian Rivers Institute. For example, some PPCPs affect immune system function or behavior, responses that risk assessments don’t currently include, she says.

“The current testing approaches are not sufficiently optimized for efficiently finding either the species, the endpoints, or the drugs that are probably of most concern,” Larsson adds. He and Kidd agree that the new pilot study’s effort to document which compounds are taken up by exposed fish is an important step in the right direction.

## Figures and Tables

**Table:** 

Compound	Use	≥ MDL in Liver	≥ MDL in Fillet
Fluoxetine	Antidepressant	yes	no
Norfluoxetine	Antidepressant	yes	yes
Sertraline	Antidepressant	yes	yes
Diphenhydramine	Antihistamine	yes	yes
Diltiazem	Antihypertensive	yes	yes
Atenolol	Antihypertensive	no	no
Metoprolol	Antihypertensive	no	no
Propanolol	Antihypertensive	no	no
Carbamazepine	Antiseizure	yes	yes
Triclosan	Antimicrobial	no	no
Galaxolide	Fragrance	no	yes
Tonalide	Fragrance	no	yes
Celestolide	Fragrance	no	no
Gemfibrozil	Lipid regulator	yes	no
1,7 Dimethylxanthine	Antispasmodic	no	no
Acetaminophen	Analgesic	no	no
Ibuprofen	Analgesic	no	no
Caffeine	Stimulant	no	no
Cimetidine	Anti–acid reflux	no	no
Codeine	Analgesic	no	no
Erythromycin	Antibiotic	no	no
Lincomycin	Antibiotic	no	no
Sulfamethoxazole	Antibiotic	no	no
Trimethoprim	Antibiotic	no	no
Tylosin	Antibiotic	no	no
Miconazole	Antifungal	no	no
Thiabendazole	Antifungal	no	no
Warfarin	Blood thinner	no	no
4-Methylbenzylidene camphor	Ultraviolet filter	no	no
Octocrylene	Ultraviolet filter	no	no
*m*-Toluamide	Insect repellant	no	no
Musk ketone	Fragrance	no	no
Musk xylene	Fragrance	no	no
Nonylphenol	Surfactant	no	no
Octylphenol	Surfactant	no	no

